# Bayesian-optimized machine learning and experimental study of Al₂O₃-CuO hybrid nanofluid thermal performance in turbulent circular tube flow

**DOI:** 10.1038/s41598-025-23785-3

**Published:** 2025-11-05

**Authors:** Praveen Kumar Kanti, H. B. Marulasiddeshi, Nejla Mahjoub Said, V. Vicki Wanatasanappan, Prabhu Paramasivam, Leliso Hobicho Dabelo

**Affiliations:** 1 Institute of Power Engineering, Universiti Tenaga Nasional, Jalan IKRAM-UNITEN, Selangor, 43000 Malaysia; 2 Department of Mechanical Engineering, SJCE, JSS Science and Technology University, Mysuru, 570006 India; 3 Department of Physics, College of Science, King Khalid University, Abha, 61413 Saudi Arabia; 4 Department of Research and Innovation, Saveetha School of Engineering, SIMATS, Chennai, 602105 Tamil Nadu India; 5 Department of Mechanical Engineering, Mattu University, Mettu, 318 Ethiopia; 6 Centre for Research Impact & Outcome, Chikara University Institule of Engineering and Technology, Chitkara University, Rajpura, 140401, Punjab, India

**Keywords:** Constant heat flux, Entropy generation, Heat transfer, Hyperparameters tuning, Numerical study, Engineering, Mechanical engineering

## Abstract

This study explores the thermal behavior of hybrid nanofluids (HNFs) composed of water mixed with equal proportions (50:50) of Al₂O₃ and CuO nanoparticles (NPs) under turbulent flow regimes. The nanofluids (NFs) are prepared in the volume concentrations range of 0–1%. Both experimental investigations and numerical simulations were carried out to evaluate the effects of NP concentration and Reynolds number (Re) on Nusselt number (Nu), friction factor, and entropy generation. Results demonstrated a marked enhancement in heat transfer with increasing NP concentration and flow rate. Notably, the use of HNFs led to a 71% reduction in total entropy generation (TEG) compared to water alone. Empirical correlations were developed to predict the Nu and friction factor accurately. Furthermore, an XGBoost machine learning model was employed to estimate thermal parameters with high precision. The model achieved an R² of 1.000 (training) and 0.991 (testing) with an MSE of 0.001 for TEG. For the friction factor, R²_training_ as 0.686 and R²_test_ as 0.916 (testing) were obtained. Nu model achieved perfect training accuracy (R² = 1.000) and strong testing performance (R² = 0.975, MSE = 29.457). These results affirm the effectiveness of XGBoost in modeling thermofluidic behavior in HNF systems.

## Introduction

 The rapid growth in industrial activity has driven a growing mandate for efficient energy utilization. As a result, continuous advancements in heat transfer technologies have emerged to meet this need^1^. However, the performance of conventional heat transfer fluids (HTFs) is still limited by their inherently low thermal conductivity (TC)^2^. To address this, Choi^3^ introduced NFs, a novel class of HTFs enhanced with NPs smaller than 100 nm. These fluids exhibit improved thermophysical properties, particularly TC, leading to better heat transfer performance. The addition of metallic or non-metallic NPs increases the surface area available for heat exchange, thereby enhancing the thermal characteristics of the base fluid^4^. Despite these improvements, the enhancement remains moderate. Recent research has shifted toward HNFs, which combine two or more different types of NPs within a base fluid to leverage their synergistic effects^5^. Studies have shown that HNFs generally outperform mono nanofluids in TC. However, both mono and HNFs can increase the fluid’s viscosity (VST), leading to higher pressure drops and friction factors. This rise in VST, particularly at higher NP concentrations, results in greater pumping power requirements and potential operational penalties^6–8^.

Recent studies have increasingly turned attention toward the thermal and hydrodynamic behavior of HNFs, which combine three different types NPs to leverage their complementary properties^9–11^. HNFs have shown considerable potential in enhancing heat transfer characteristics. For instance, Ekiciler^12^ reported improved thermal performance with NFs when used in pipes with corrugated walls. Numerical investigations have also offered detailed insights into heat transfer parameters such as the Nu, friction factor, temperature profiles, and velocity fields^13,14^. Kanti et al.^15^ reported that 1 vol% Al_2_O_3_-TiO_2_ HNF enhances VST by 15.8% and boosts the Nu by 70.4% compared to water at 30 °C.

Shahsavar et al.^16^ performed a numerical study on natural convection in a concentric annulus using a HNFs composed of Fe₃O₄ and carbon nanotubes, highlighting that higher concentrations increased both thermal and frictional entropy generation. Boruah et al.^17^ evaluated heat transfer and entropy generation in a microchannel using a two-dimensional, laminar, incompressible flow of Al₂O₃ NFs. They observed a consistent rise in average Nu with increasing Re at all NF concentrations. Seyyedi et al.^18^ examined the effects of a magnetic field on heat transfer and entropy generation in a hexagonal cavity filled with copper NF. Their results showed that increasing the aspect ratio enhanced mean Nu valueas well as entropy generation.

Chinakwe et al.^19^ performed a numerical analysis to evaluate the influence of pipe inclination, Reynolds number, and nanoparticle concentration on heat transfer characteristics, hydraulic response, and entropy production for Al₂O₃ nanofluids in turbulent flow through a smooth circular aluminum tube. Their calculations demonstrated that a + 45° tilt resulted in the most significant thermal variances among the evaluated orientations, highlighting the substantial influence of gravitational and geometric forces in inclined conduits. Sundar et al.^20^ and Kanti et al.^21^ conducted complementary tests that showed that the contribution of frictional irreversibility to overall entropy creation is rather minor. This suggests that thermal processes are the main cause of exergy losses in normal operating settings. Developing high-performance heat transfer fluids is still a top priority for applications in electronics cooling, energy conversion systems, and transportation thermal management, where even little improvements in thermal properties can have a big impact on the system as a whole. There is a lot of interest in hybrid nanofluids, but there are not many good datasets on the combined thermal performance and entropy-generation properties of Al₂O₃-CuO hybrids across different operating windows and mixing ratios. The current study aims to fill this gap by systematically investigating heat transfer responses and thermodynamic irreversibilities in Al₂O₃-CuO hybrid nanofluids. The goal is to provide insights for the engineering of next-generation heat transfer fluids that exhibit enhanced thermal efficiency and minimized entropy generation under pertinent flow and boundary conditions.

This study investigates the convective heat transfer and flow behavior of water-based Al_2_O_3_-CuO (50:50) HNFs inside a tube having a circular cross-section under constant heat flux of 8625 Wm^−2^. The test nanofluids were synthesized through the process of varying the concentration in the range of 0 to 1 vol%. Computational fluid dynamics (CFD) simulations were conducted using ANSYS software to validate experimental data and gain a deeper understanding of the underlying physics at Re ranging from 7000 to 26,444. A modern and ensemble-based machine learning approach extreme gradient boosting was used for developing the forecasting models employing the experimental data. This research aims to provide accurate predictions for these HNFs, ultimately contributing to a better understanding of NF thermodynamics, the development of predictive tools for thermal system optimization, and the exploration of potential applications of HNFs in various engineering fields. These advancements have the potential to drive innovation and enhance thermal management technologies across different industries.

## Materials and methods

### Preparation of nanofluid

The test nanoparticles (Al₂O₃) were less than 30 nm dia. in size, whilst CuO nanoparticles measured approximately 13 nm^22^. A Wensar digital balance was used to accurately weigh the required quantities of NPs^22^. The NFs used in the study were synthesized employing two-step synthesis method. Spherical Al₂O₃ NPs and a 1:1 mixture of Al₂O₃ and CuO NPs were added to 60 mL of water to prepare various concentrations of mono NF and HNF, respectively. Prior to NPs dispersion, sodium dodecylbenzene sulfonate (SDBS) was introduced as a surfactant (20% by weight for Al₂O₃ NFs and 30% for HNFs), relative to the respective NP concentrations. The mixture was stirred using a REMI 2MLH magnetic stirrer at 1000 rpm for one hour. This was followed by ultrasonication (400 W, 24 kHz) for four hours to minimize agglomeration and ensure stable, long-term dispersion of nanoparticles in the base fluid^22^.

### Thermophysical properties

The main thermophysical characteristics are listed in Table 1. kindly to our published paper for more details on the thermophysical properties of test NFs^22^. Following equations were employed to estimate specific heat (SH) and density of NFs^25,26^.

1 $$\:{\rho\:}_{nf=\:}{\rho\:}_{np1\:}\left({\varnothing\:}_{1\:\:}\right)+{\rho\:}_{np1\:}\left({\varnothing\:}_{2\:\:}\right)+\:{\rho\:}_{bf}(1-{\varnothing\:}_{1\:}-{\varnothing\:}_{2\:\:})$$

2 $$\:{\left({\rho\:C}_{p}\right)}_{nf}=\:{\left({\rho\:C}_{p}\right)}_{np1}\left({\varnothing\:}_{1\:\:}\right)+\:{\left({\rho\:C}_{p}\right)}_{np2\:}\left({\varnothing\:}_{2\:\:}\right)+{\left({\rho\:C}_{p}\right)}_{bf}(1-{\varnothing\:}_{1\:}-{\varnothing\:}_{2\:\:})$$

Where, np1 and np2 refers to the Al_2_O_3_ and CuO nanoparticles, respectively.

nf, $\:{\varnothing\:}_{1\:\:}$, and $\:{\varnothing\:}_{2\:\:}$refers to the nanofluid, volume conc. of Al_2_O_3_ and CuO NPs, respectively. $\:\rho\:$ and $\:{C}_{p}$ are density and SH, respectively.

**Fig. 1:**
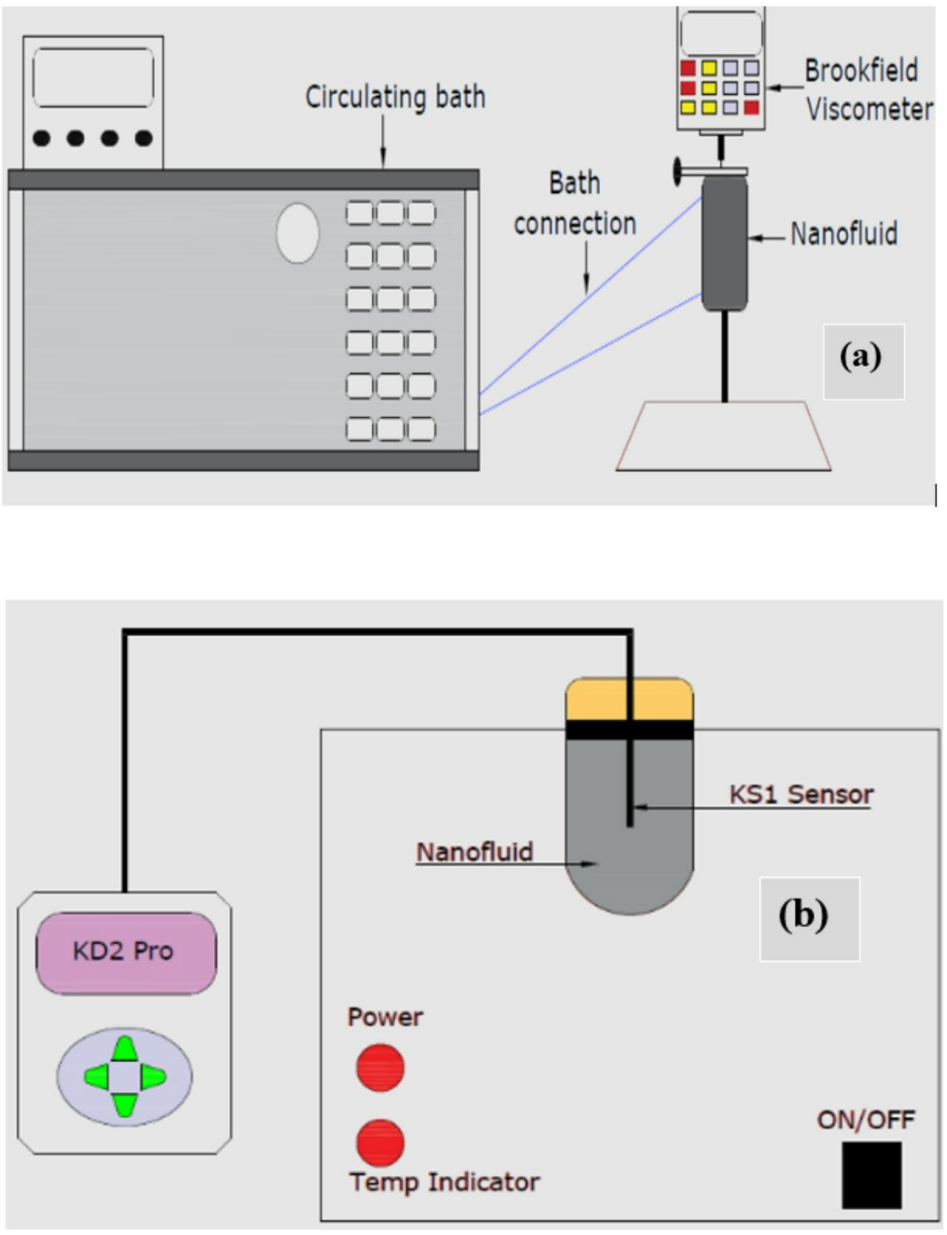
Instruments used to measure nanofluid (**a**) viscosity and (**b**) thermal conductivity.

The TC of the HNFs was measured using the Decagon Devices Inc., USA make KD2 Pro thermal analyzer, a standard instrument in nanofluid studies. It was calibrated by employing glycerol prior to testing. Temperatures were observed between 30 and 60 °C, with five values averaged after 20-minute spacing. VST was measured with an LVDV-II Pro Brookfield programmable viscometer across the same temperature range, with values collected every 15 min. The mean values from each set of measurements were used for analysis^8,22^. Figure 1 depicts the schematic diagram of the instruments used in this work to measure nanofluid viscosity and TC.

**Table 1:** Thermophysical properties of nanoparticles.

Specifications	CuO^22,23^	Al_2_O_3_^21–24^
Purity	99.5	99.8
Particle size (nm)	13	30
⍴, kg m^−3^	6300	3970
$\:{\text{C}}_{\text{p}}$, J kg^−1^ K^−1^	535	773
k, W m^−1^ K^−1^	76.5	36

### Test facility

**Fig. 2:**
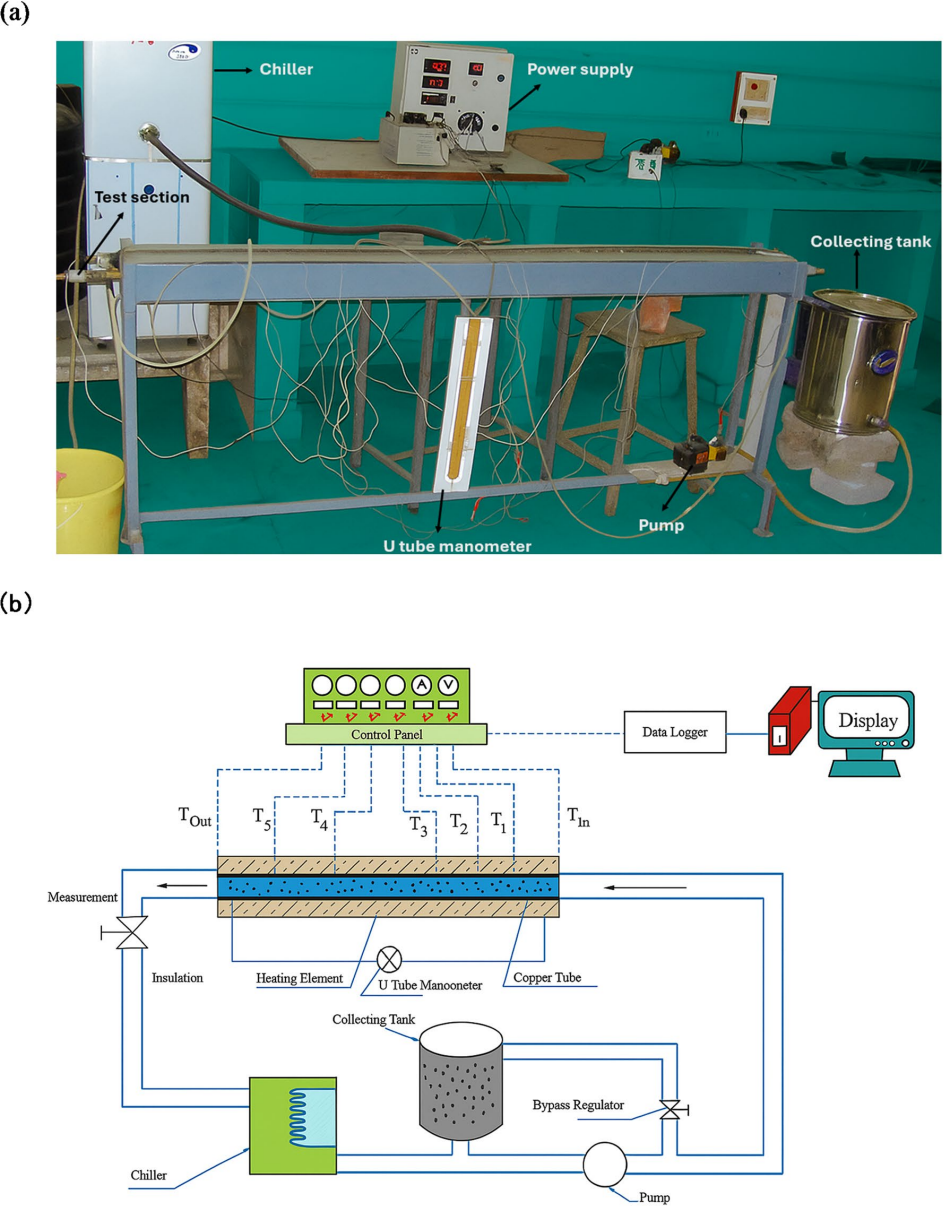
(**a**) Actual experimental setup. (**b**) Schematic of the experimental setup.

Figure 2a shows the actual experimental setup, while Fig. 2b presents its schematic, comprising a copper test section, a nichrome heating element, a circulation pump, a chiller, a stainless steel (SS) collector tank, and a U-tube manometer^15^. The test section is a copper tube with an inner diameter of 16 mm, an outer diameter of 19 mm, and a length of 1500 mm^15,21^. Precision K type thermocouples were installed at the inlet and outlet of the tube, as well as at intervals of 250 mm along its length, and brazed at the specified positions to ensure accurate temperature measurements^25^. A 1500 W nichrome wire heater (Make: Omega, USA) was used to uniformly heat the test section. Temperature measurements were taken with a Countronics (India) data recorder linked to the thermocouples. The entire tube had been insulated with asbestos rope to minimize heat loss. The working fluid had been circulated through a 0.02 m³ SS collection tank utilizing a 0.5 hp pump (model: Kirloskar).

A base fluid temperature of 30 °C was maintained at all flow rates with a tolerance of ± 1 °C, using a 2000 W water chiller^21^. Fluid pressure was monitored via a U-tube manometer, which is essential for evaluating the thermohydraulic performance of the system^26^.

### Data analysis

The heat transfer coefficient (h) and Nu were estimated by using Eqs. (3) and (4), employing experimental data^15,21,25,26^:

3a $$\:\text{h}\:=\frac{\text{Q}}{{\text{A}}_{\text{s}}({\text{T}}_{\text{s}\:}-{\text{T}}_{\text{b}})},$$

Where, Q = V$\:\times\:$I (heat supplied) in Watts, (3a)

3b $${{\text{A}}_{\text{s}}}=\pi {\text{DL}},$$

3c $$\:{\text{T}}_{\text{b}}=\frac{{\text{T}}_{\text{i}}+{\text{T}}_{\text{o}}}{2},$$

3d $$\:\:{\text{T}}_{\text{s}}=\frac{{\text{T}}_{1}+{\text{T}}_{2}+{\text{T}}_{3}+{\text{T}}_{4}+{\text{T}}_{5}}{5}$$

4 $$\:\text{N}\text{u}\:=\frac{\text{h}\:\text{D}}{\text{k}}$$

For internal flow through a tube, it is essential to provide reliable reference correlations to validate the experimentally and computationally obtained results for ‘f’ and ‘Nu’. The Nu for water, derived from both experiments and CFD simulations, was compared with the standard Dittus–Boelter correlation^24^, as presented in Eq. (5)^15,21^.

5 $$Nu\,=\,0.023R{e^{0.8}}P{r^{0.4}}$$

The measured pressure drop (*∆p*) values were used to calculate the Darcy friction factor using Eq. (6). The obtained results were then compared with standard correlations provided by Petukhov^27^ and Blasius^28^, as represented in Eqs. (7) and (8), respectively^15,26^.

6 $$\:\text{f}\:=\frac{\varDelta\:p}{\left(\frac{L}{D}\right)\:\left(\frac{\rho\:{V}^{2}}{2}\right)}$$

7 $$\:{\text{f}=\:(0.79\:{\text{l}\text{o}\text{g}}_{n}\:\text{R}\text{e}\:-\:1.64)}^{-2} \,\text {subjected to 3000} < Re < 5 \:\times\: 10^{6}$$

8 $$\:\text{f}=\frac{0.316}{{\text{R}\text{e}}^{0.25}}$$

Equation (9) denotes the TEG (S_gen, T_) of HNF as the sum of heat transfer (S_h, t_) and frictional entropy (S_f, t_) generation^15,20,25,26^.

9 $${{\text{S}}_{{\text{gen}},{\text{ T}}}}={\text{ }}{{\text{S}}_{{\text{h}},{\text{ t}}}}+{\text{ }}{{\text{S}}_{{\text{f}},{\text{t}}}}$$

TEG for the tube was calculated by using the following expression (10)^15,20^.

10 $$\:{\text{S}}_{\text{g}\text{e}\text{n},\:\:\text{T}}=\frac{{\text{Q}}^{2}}{{\uppi\:}\:\text{k}\:\text{L}\:{\text{N}\text{u}\:\text{T}}_{\text{i}\text{n}}\:{\text{T}}_{\text{o}\text{u}\text{t}\:\:}}\:+\:\frac{8\:{\text{L}\:\text{m}}^{3}\text{f}\:}{{\:{\uppi\:}}^{2}\:{{\uprho\:}}^{2}{\text{D}}^{5}\left({\text{T}}_{\text{o}\text{u}\text{t}\:-}{\text{T}}_{\text{i}\text{n}\:}\right)}\:\text{l}\text{n}\:\left(\frac{{\text{T}}_{\text{o}\text{u}\text{t}\:\:}}{{\text{T}}_{\text{i}\text{n}\:\:}}\right)$$

The Bejan number (*Be*), is introduced to explore the effect of S_h, t_ as well as friction S_f, t_ over the TEG^15,26^

11 $$\:\text{B}\text{e}=\frac{{S}_{h,\:t}}{{\text{S}}_{\text{g}\text{e}\text{n},\:\:\text{T}}\:}$$

### Numerical model

**Fig. 3:**
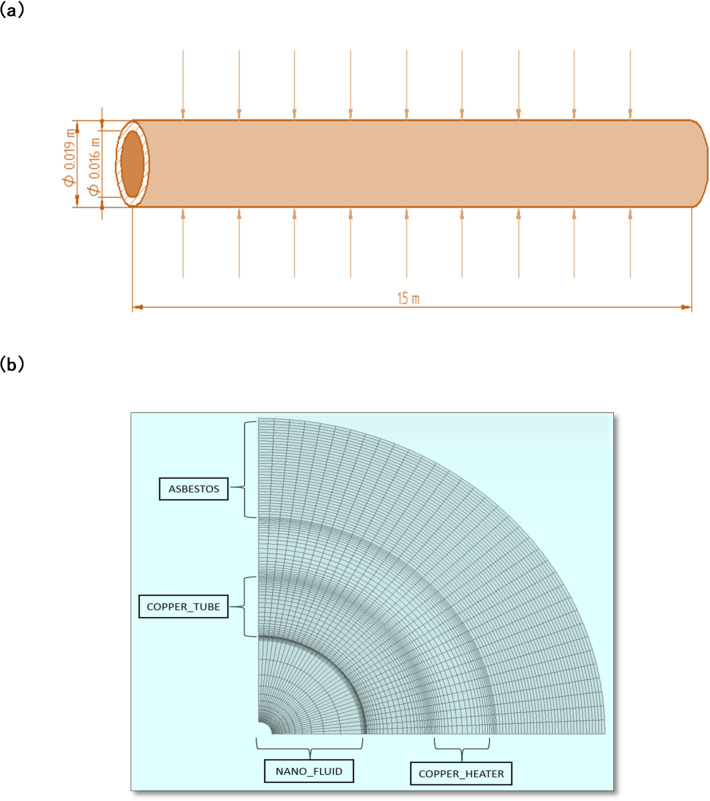
(**a**) Schematic diagram of the test section under constant heat flux. (**b**) Copper tube model showing mesh.

The testing setup was replicated in a specialized computational domain to model the thermal and hydrodynamic characteristics of the system. At the outlet of the tube, an exit pressure condition was applied, while the inlet was assigned varying fluid velocities based on experimental flow rates^21^. The inlet fluid temperature was maintained at 30 °C, and the outer wall of the test section was subjected to a constant heat flux of 8625 W/m². To ensure a fully developed flow, a no-slip boundary condition was applied, and the inlet and outlet sections were extended to five times the inner diameter of the tube^21,25^. The extended walls were treated as adiabatic, with zero heat flux^26^. Figure 3a and b present the geometric model and corresponding mesh used in the simulations. The geometry was meshed with structured hexahedral elements and refined iteratively to validate the CFD results using water.

A realizable *k-ε* turbulence model with enhanced wall treatment and full *Y*^*+*^ treatment was adopted for this study^26^. The *Y*^*+*^ values across the computational domain were maintained below 1, ensuring adequate near-wall resolution^23,29^. The simulations were performed using ANSYS Fluent, employing the finite volume method to solve the governing equations for turbulent forced convection. Hexahedral meshes were used for both fluid and solid regions to improve numerical accuracy and stability^21^. Turbulent intensity (*I*) was estimated using the empirical correlation:

12 $$\:I=0.16\text{R}{\text{e}}^{-1/8}$$

For the numerical study, the following assumptions were made: the nanofluid behaves as a steady, incompressible, and Newtonian fluid; gravitational effects were neglected; and the thermophysical properties of the nanofluid were assumed constant^26,30,31^. The governing equations used in this simulation can be found in our previously published article^32^.

### Extreme gradient boosting

The evolution of XGBoost from conventional decision tree methodologies represents a pivotal breakthrough within the domain of artificial intelligence and machine learning algorithms^29^. A thorough comprehension of XGBoost requires an examination of the basic models that preceded and shaped its architecture^33^. The creation of XGBoost was motivated by the urgent need for an effective and scalable gradient boosting system. Current techniques, despite their widespread use, are limited by performance limitations associated with computing speed and scalability^34^. In response, Chen developed XGBoost to address these technological challenges and enhance model performance. Its sustained superiority in international machine learning competitions, such as those hosted by Kaggle and KDD Cup demonstrates its practical effectiveness and scholarly importance^33^.

The XGBoost technique is fundamentally based on the decision tree, a core supervised learning framework initially introduced by Quinlan in 1986 for application in regression and classification tasks. This framework methodically divides the feature space into subregions by threshold-based divisions of input variables, resulting in a hierarchical tree structure. In this framework, the root node represents the primary division of the dataset, internal nodes denote further recursive divisions, and terminal nodes (leaves) embody the model’s output^35^. In regression analysis, each leaf generally yields the mean response value of the data included within that node. In classification issues, the leaf node often designates the class label that is most dominant among the related occurrences. In modern research applications, the efficacy of XGBoost is enhanced with the use of Bayesian optimization methods. These approaches are employed to systematically investigate the hyperparameter space, providing a probabilistic model-based search mechanism that emphasizes attractive combinations.

### Bayesian optimization

Bayesian optimization (BO) is an optimization approach for optimizing objective functions that are difficult to evaluate and lack analytical expressions. Unlike exhaustive methods such as grid search, which evaluate all combinations in a predefined hyperparameter space^36,37^, BO leverages probabilistic models like Tree-structured Parzen Estimators or Gaussian Processes (GPs) or to predict the performance of unseen configurations based on prior evaluations. Its structure involves two fundamental components: a surrogate model that approximates the unknown objective function and an acquisition function that guides the selection of the next set of hyperparameters to evaluate. The surrogate model is updated iteratively as new results are obtained, thereby improving the optimization process with each evaluation^38,39^.

The process begins with an initial set of hyperparameter evaluations, often chosen at random. The surrogate model then estimates the likely performance of new configurations, and the acquisition function determines which point in the hyperparameter space to sample next, balancing exploration of uncertain regions and exploitation of known good regions. This results in a far more efficient search strategy, especially useful in scenarios where model training is computationally intensive, such as with deep learning or ensemble methods like XGBoost^40,41^. Compared to grid search and random search, BO offers superior sample efficiency, often requiring significantly fewer evaluations to locate optimal or near-optimal configurations. However, its complexity and computational overhead for surrogate model fitting and acquisition function optimization make it less suitable for problems with very low computational costs or extremely high-dimensional parameter spaces^42,43^. It is particularly effective when function evaluations are expensive and when model performance varies smoothly with hyperparameters. However, it may struggle with highly non-smooth or discontinuous response surfaces. BO is thus ideal for applications involving costly model training or simulation, where each iteration is resource-intensive, and a minimal number of evaluations is desirable^44^.

## Results and discussion

### Stability

**Fig. 4:**
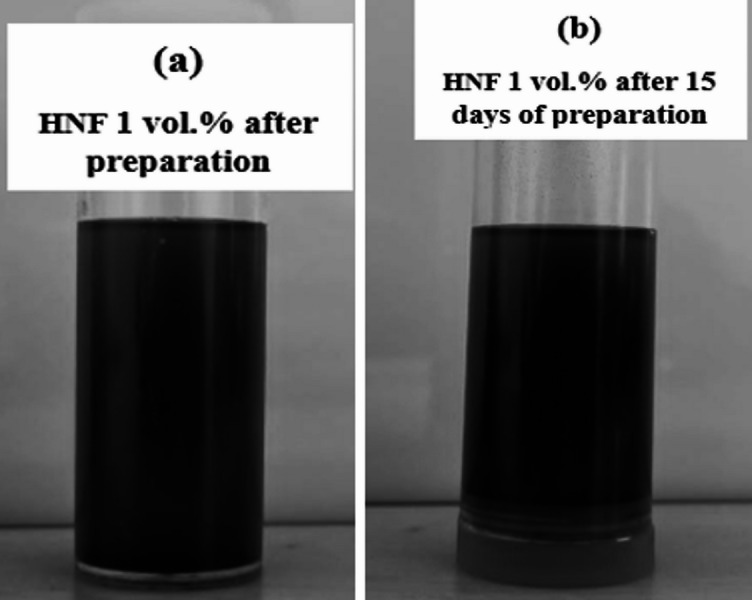
Photograph of Al₂O₃–CuO (50:50) hybrid nanofluid immediately after (**a**) preparation and (**b**) 15 days, illustrating its colloidal stability over time.

Zeta potential is a key indicator of NF stability, with values above $\:\pm\:$30 mV generally reflecting strong electrostatic repulsion among particles, thereby preventing aggregation and enhancing dispersion^15,22^. Values below this threshold suggest weaker repulsive forces and a higher likelihood of particle agglomeration and sedimentation. Table 2 presents the measured zeta potentials for both NFs^22,25^. As NP concentration increases, stability tends to decline due to increased inter-particle interactions that promote clustering. Al₂O₃ NFs characteristically exhibit higher stability in comparison to their Al₂O₃–CuO hybrid counterparts. This is attributed to the uniform particle size and surface characteristics of Al₂O₃, which support better dispersion. In contrast, HNFs combine particles of differing sizes and surface charges, making stable suspension more challenging. As a result, they often show lower zeta potential values, indicating reduced electrostatic repulsion and lower stability relative to mono nanofluids^22^. Figure 4 presents the photographs of the prepared nanofluid immediately after preparation and after 15 days. It is clearly observed that there is no visible sedimentation in either case, indicating good dispersion and excellent long-term stability of the nanofluid. This observation is further supported by the zeta potential values presented in Table 2, which confirm the stable nature of the suspension.

**Table 2:** Zeta potential (mV) values for both the nanofluids.

Vol%	Al_2_O_3_ NF		Al_2_O_3_-CuO HNF	
	After preparation	After 2 weeks	After preparation	After 2 weeks
0.1	41.1	39.6	39.2	37.2
0.3	40.6	39	37.1	36.8
0.5	39.1	37.4	35.3	34.4
0.75	37.8	36.5	34.5	33.2
1.0	35.1	34.2	33.9	32.8

### Thermophysical properties

**Fig. 5:**
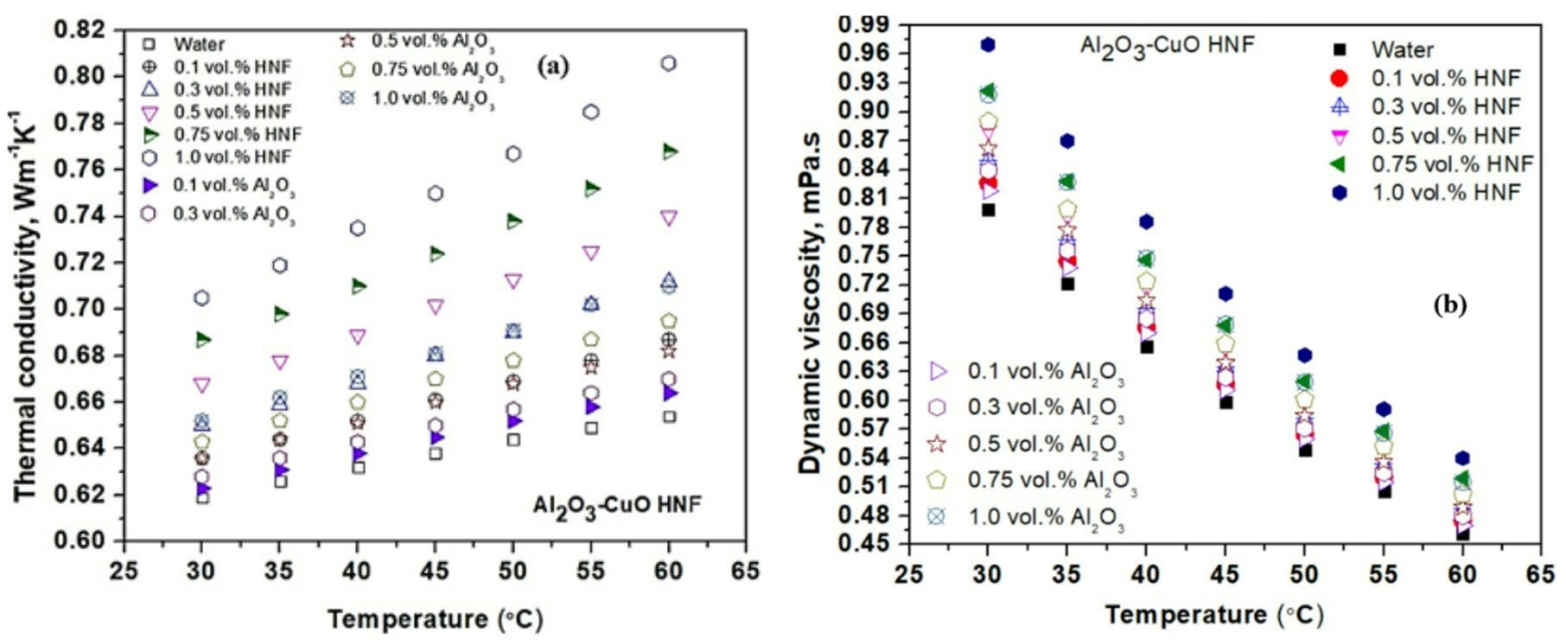
Variation of nanofluids (**a**) TC and (**b**) viscosity with temperature.

Figure 5a shows that TC of both mono and HNFs increases with temperature and concentration. Elevated temperatures reduce base fluid VST, enhancing NP mobility and phonon transport, which in turn boosts TC^15^. Improved dispersion at higher temperatures also promotes uniform heat conduction. Increasing concentration introduces more thermal pathways, while stronger inter-particle interactions at higher loads enhance alignment and stability, further improving TC. The Al₂O₃-CuO HNF outperforms mono Al₂O₃ NF due to the synergistic effect of combining two distinct NPs. Smaller Al₂O₃ particles offer higher surface area, while larger CuO particles bridge thermal gaps. This pairing reduces phonon scattering, minimizes agglomeration, and enhances dispersion, resulting in superior thermal performance. The maximum TC values of 0.654, 0.71 and 0.806 mPa.s for water, Al_2_O_3_ (1 vol%) and HNF (1 vol%) found at 60 °C, respectively.

Figure 5b illustrates the variation in VST of both mono and HNFs with respect to temperature and concentration. VST increases with concentration due to intensified interactions between base fluids and NPs, which raise the flow resistance caused by higher internal friction resistance to flow. Conversely, VST decreases with rising temperature, as increased thermal energy weakens intermolecular forces in the base fluid, allowing for smoother flow^45^. The Al₂O₃-CuO HNF exhibits higher VST than the Al₂O₃ NF. This is attributed to the complex interaction dynamics introduced by combining NPs of different sizes and properties. The mixed NPs system creates a more intricate internal structure, increasing flow resistance and resulting in greater overall VST compared to mono nanofluids. The maximum VST values of 0.798, 0.918 and 0.97 mPa.s for water, Al_2_O_3_ (1 vol%) and HNF (1 vol%) found at 30 °C, respectively.

### Mesh independence test

Table 3 compares the Nu obtained from CFD simulations using three different mesh sizes with experimental results and the Dittus–Boelter correlation (Eq. 5) at an inlet temperature of 30 °C across a range of Re. Boundary layers in both fluid and solid domains were carefully refined to capture accurate thermal and flow behavior. Structured hexahedral meshing was carried out in three iterations to validate CFD predictions against base fluid data. The deviations between CFD-predicted Nu and the Dittus–Boelter correlation (Eq. 5) were 3.12, 1.83, and − 3.30% for the first, second, and third mesh cases, respectively. All well within the commonly accepted deviation limit of ± 10% for heat transfer studies^15^. The second mesh was selected for further simulations as it offered the optimal trade-off between accuracy and computational efficiency minimizing deviation while avoiding unnecessary computational cost.

Additionally, the maximum deviation between experimental Nu and Eq. 5 was 2.33%, and 3.45% between CFD and experimental Nu was further confirming the consistency of the numerical and experimental methodologies. These results validate the mesh strategy and confirm the reliability of the adopted approach.

**Table 3:** Nusselt number of water from CFD (three mesh sizes), experiments, and Dittus–Boelter correlation at 30 °C for various Reynolds number.

Reynold number (Re)	Nusselt number obtained from				
	Mesh 1 (1,706,718 elements)	Mesh 2 (2,268,121 elements)	Mesh 3 (2,381,491 elements)	Dittus–Boelter correlation (Eq. 5)	Experimental
8263	60	61	62	60	60
12,463	86	87	88	86	84
16,617	111	113	114	113	111
20,772	130	133	134	134	132
24,926	147	149	151	150	149
27,638	160	162	164	165	162

### Heat transfer

**Fig. 6:**
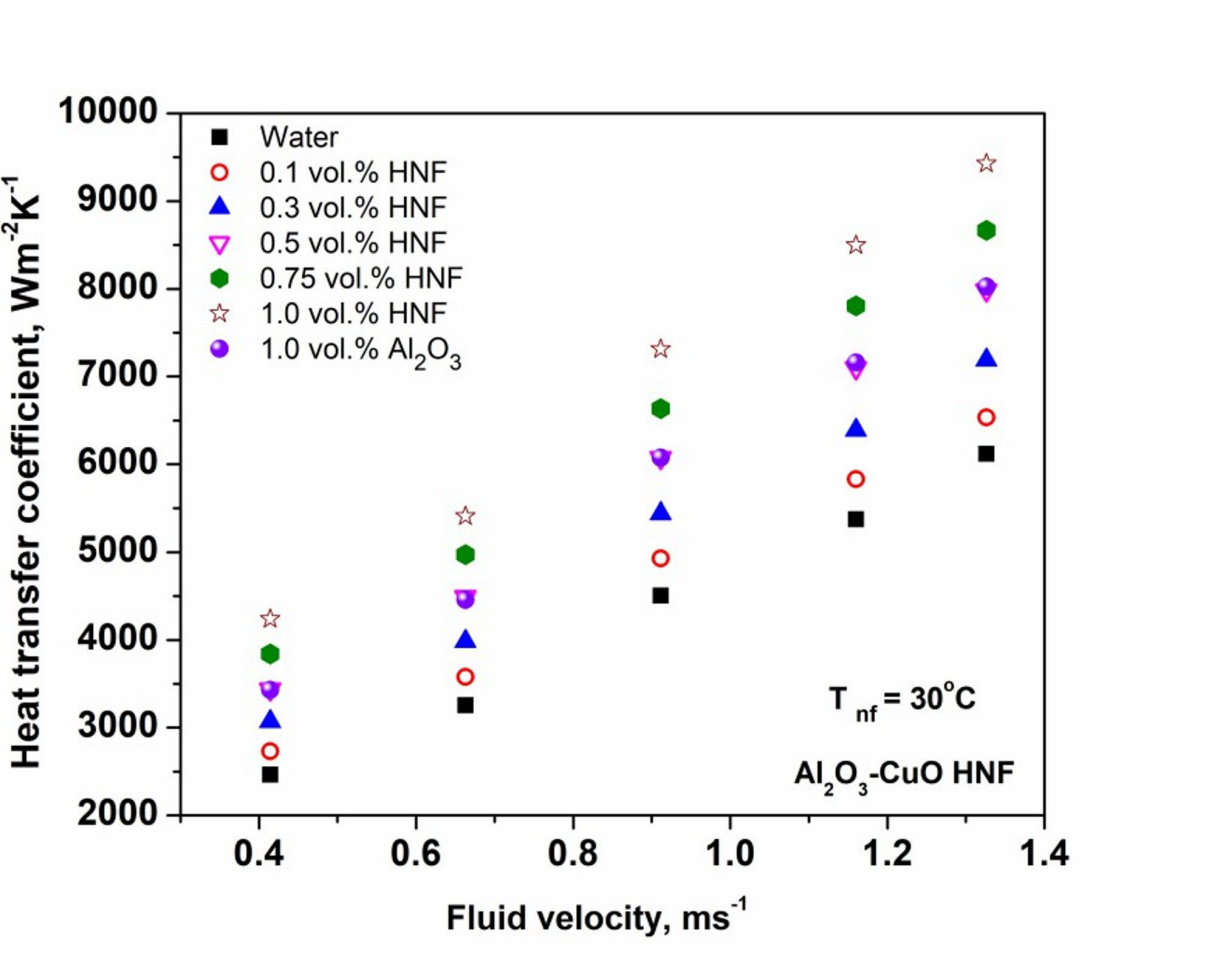
Variation of heat transfer coefficient with fluid velocity of nanofluid.

Figure 6 presents the effect of fluid velocity on the experimentally measured heat transfer coefficient (HTC). At all velocities, the HTC of the HNF exceeds that of both Al₂O₃ NF and the base fluid (water). Specifically, the HNF shows enhancements of 72 and 6.82% in HTC at 1.0 and 0.1 vol%, respectively, in comparison with water. The peak and minimum HTC improvements achieved by the HNF were 23.6 and 17.5%, respectively. The higher values is the result of raised TC and intensified Brownian motion of NPs, which promote enhanced mixing in core flow regions as well as near-wall region^15^. Additionally, the combined presence of CuO and Al₂O₃ NPs improves thermal transport due to synergistic particle interactions and delayed thermal boundary layer development^46,47^.

**Fig. 7:**
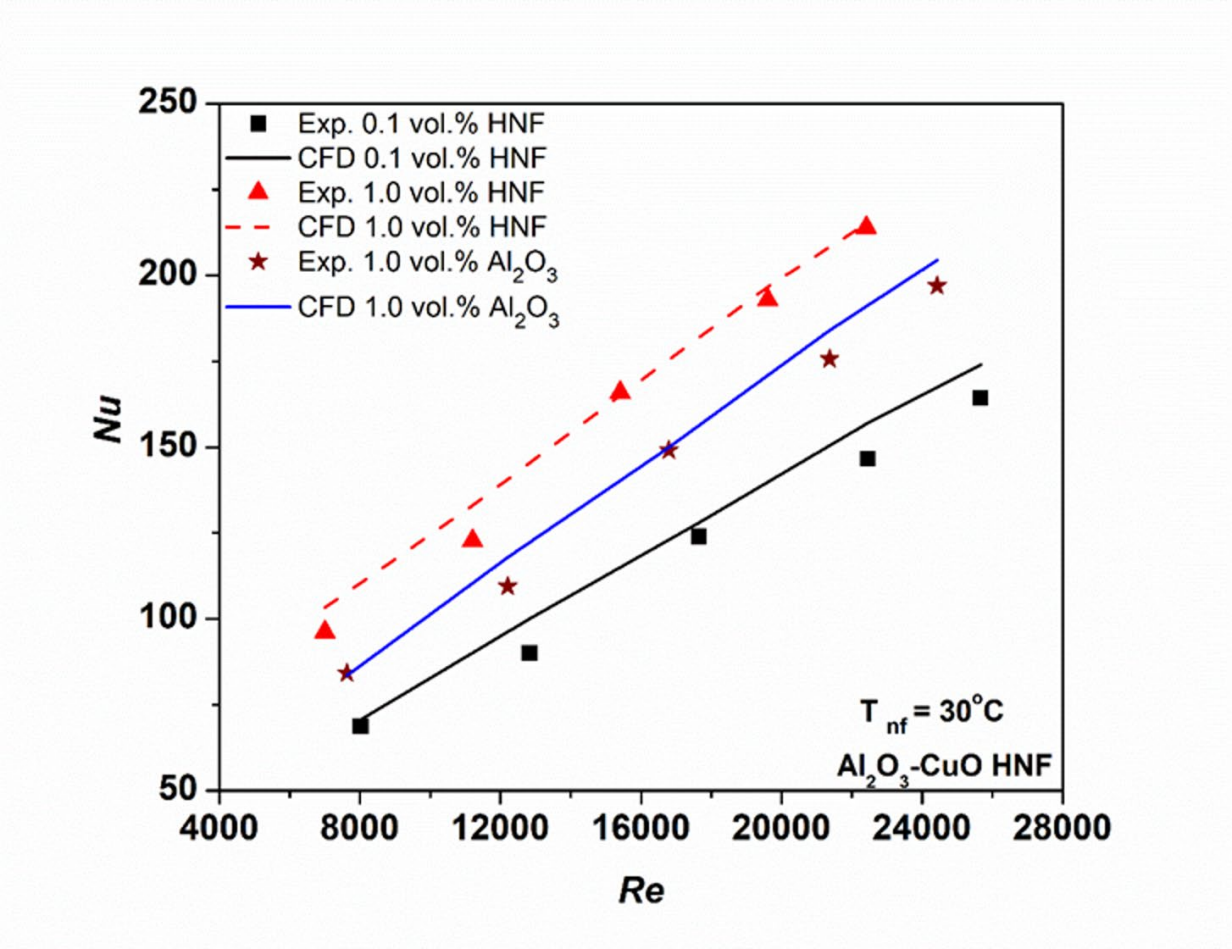
Variation of hybrid nanofluid Nusselt number with Reynolds numbers.

Figure 7 illustrates the variation of the Nu with Re for different NFs. The results show a clear increase in Nu with rising Re and concentration for both fluids, with HNFs consistently outperforming Al₂O₃ NFs at the same Re. The higher value is the result of intensified migration of particles and Brownian motion, that improve the thermophysical behavior of the hybrid suspension^26,48^. At 1.0 vol%, the maximum Nu enhancement of HNF over Al₂O₃ NF reaches 14.3%. Figure 7 further compares CFD and experimental Nu values across varying Re and concentrations. The close agreement between numerical and experimental results confirms the accuracy and reliability of the computational model.

### Pressure drop and friction factor

**Fig. 8:**
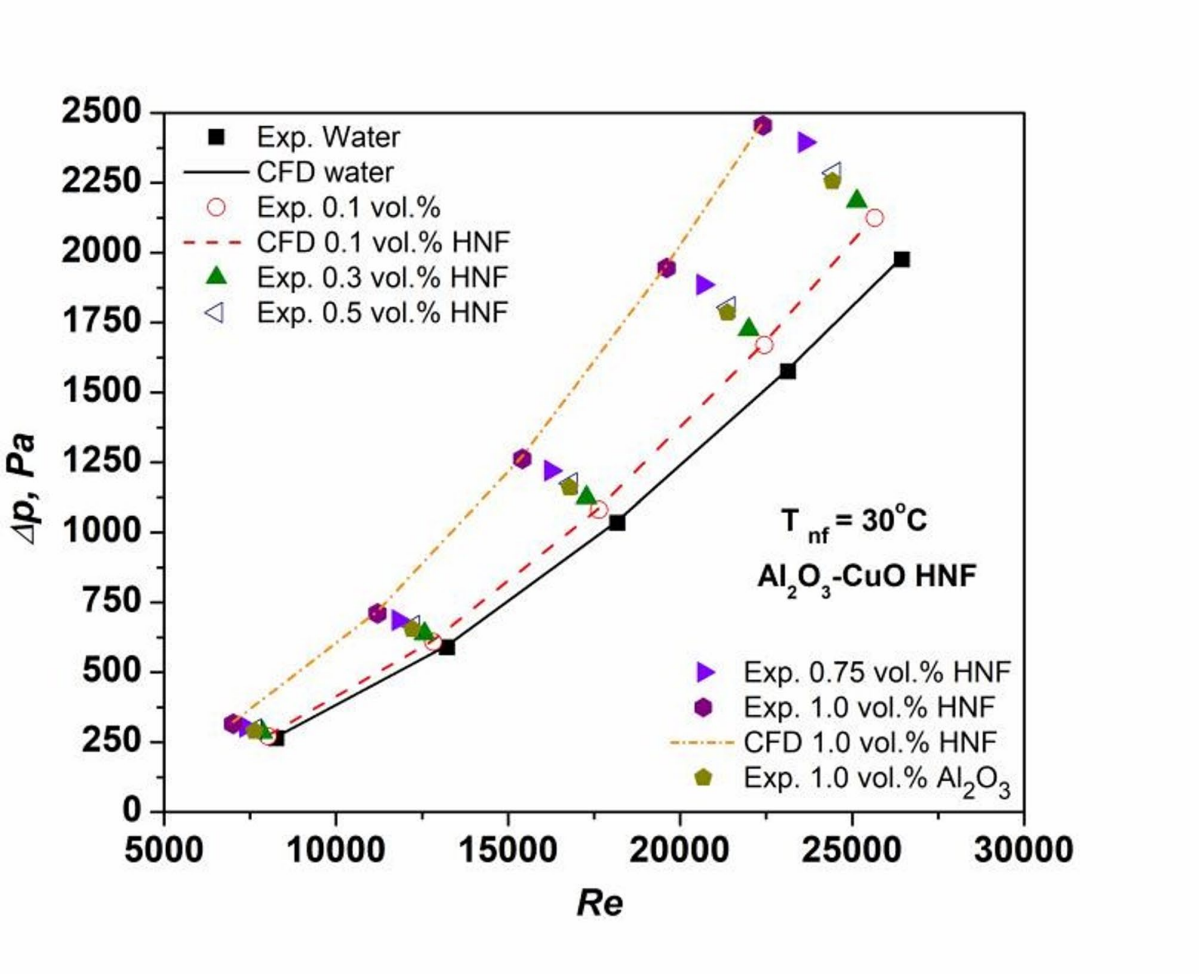
Variation of pressure drop of nanofluids with Reynolds number.

Figure 8 illustrates the variation of pressure drop (*∆p*) with Re for the tested fluids. As expected, *∆p* increases significantly with rising Re across all cases. For example, water exhibits a *∆p* rise from 264 to 1975 Pa as Re increases from 8263 to 26,444. NFs, particularly at higher concentrations, show even greater *∆p* due to elevated VST and enhanced turbulence caused by NP interactions^16,20^. The *∆p* of HNFs is notably greater to Al₂O₃ NFs, primarily attributed to inclusion of CuO NPs, which contribute to increased density and viscous shear forces^22^. At 1.0 vol%, the maximum *∆p* enhancement for HNF reaches 24.3% compared to water, while the minimum at 0.1 vol% is 2.3%. For Al₂O₃ NFs, the corresponding maximum and minimum increases are 14.2 and 9.8%, respectively. These findings confirm the influence of NP type and concentration on flow resistance.

**Fig. 9:**
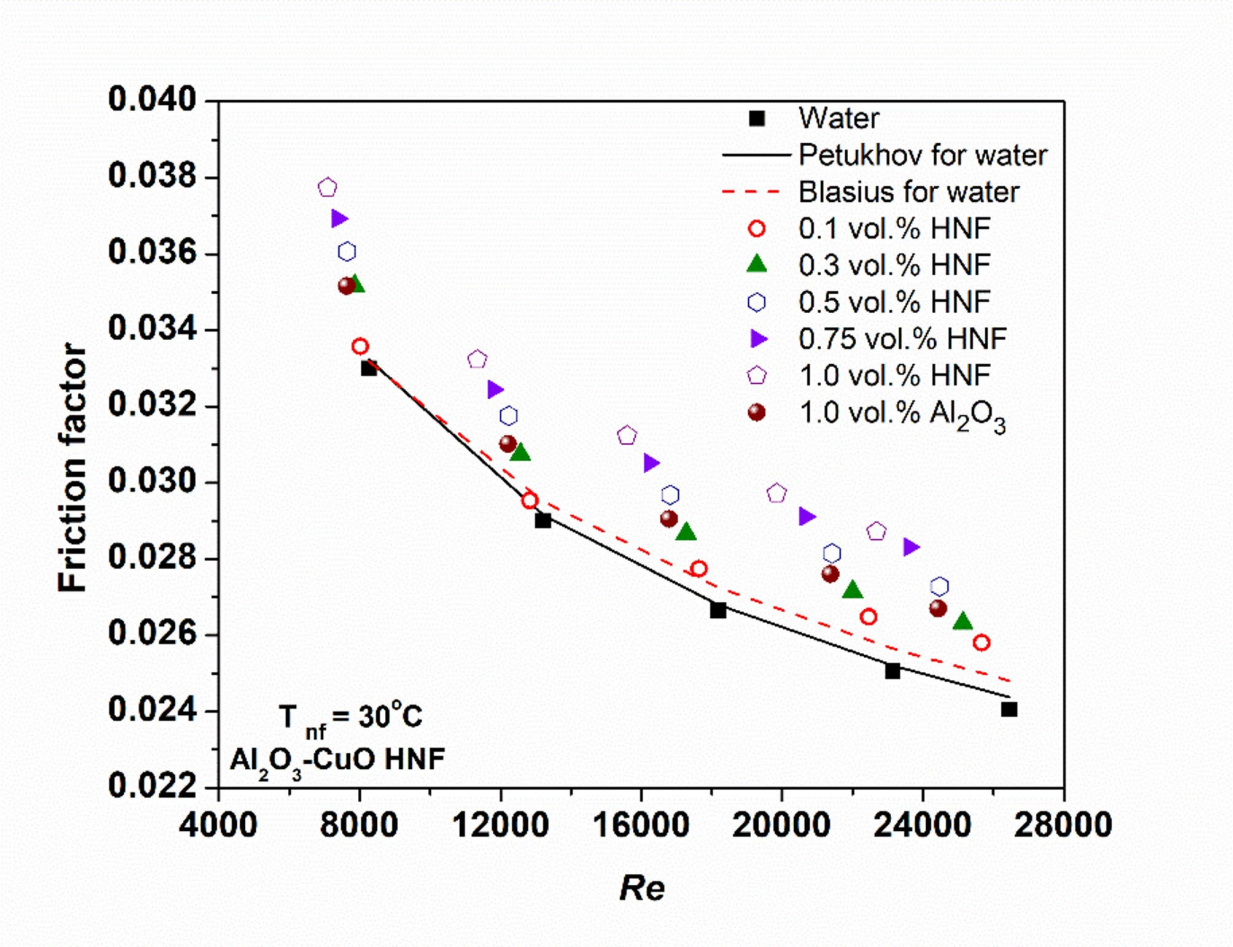
Friction factor variation of nanofluids with Re.

A key limitation of using NFs in internal flow systems is the associated increase in the friction factor, which leads to higher flow resistance and pumping power. To validate the calculated friction factor for the base fluid, results were compared with standard correlations by Petukhov^27^ and Blasius^28^. As shown in Fig. 9, the experimental friction factors for water deviate by only 1.8 and 3.1% from the Petukhov and Blasius predictions, respectively, confirming the accuracy of the methodology. Friction factors for NFs were computed using Eq. (6) and are also presented in Fig. 9. Results indicate that the friction factor increases with concentration, due to elevated VST and inter-particle interactions, but decreases with increasing Re, consistent with established trends^15,20,26^. HNFs exhibit higher friction factors than Al₂O₃ NFs, primarily due to the incidence of CuO NPs, that increase fluid density, VST, and pressure drop, collectively intensifying flow resistance^15^.

**Fig. 10:**
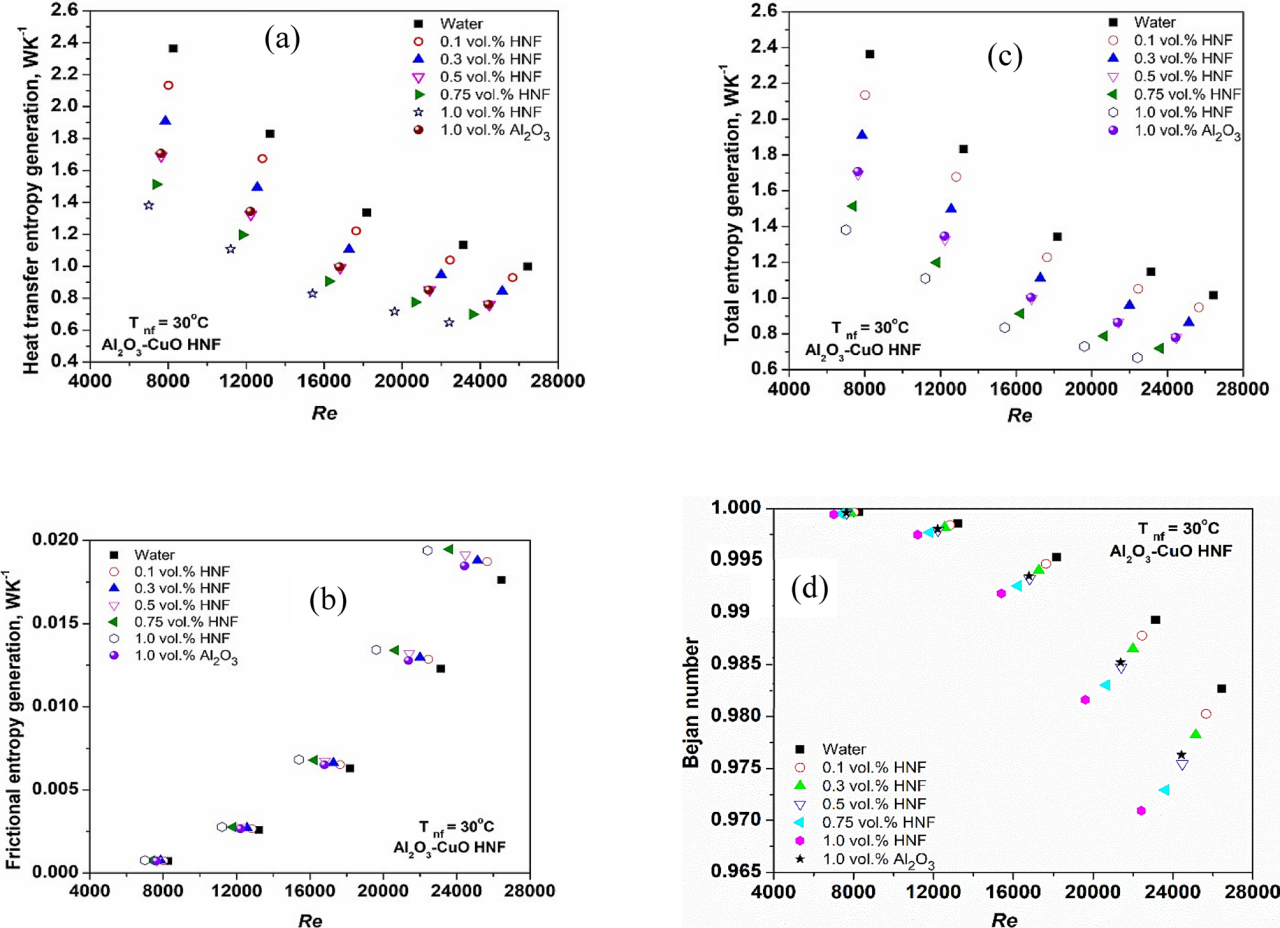
Variation of hybrid nanofluids (**a**) heat transfer vs. Re (**b**) frictional vs. Re (**c**) TEG vs. Re (**d**) Bejan number vs. Re.

Figure 10a illustrates how entropy generation associated with heat transfer in the HNF varies with Reynolds number (Re). The findings reveal that increasing either Re or nanoparticle concentration results in greater entropy generation from heat transfer. This trend can be attributed to stronger convective effects at higher Re values and intensified thermal interactions at elevated particle concentrations, particularly when the fluid–wall temperature gradient is large^47,49^. In Fig. 10c, it is evident that the total entropy generation (TEG) for both water and nanofluids is more pronounced at lower Re and reduced concentrations. These results align with the observations of Sundar et al.^20^, who also reported diminished entropy generation related to heat transfer when nanofluids were employed in place of pure water.

Figure 10b presents the generation of frictional entropy, which rises with both Re and concentration, mainly due to increased VST and pressure drop^15,20,50^. However, as illustrated in Fig. 10c, TEG tends to decrease at both higher Re and concentrations. Notably, the use of HNFs resulted in a 71% reduction in TEG compared to using water alone. This is because the contribution of frictional entropy generation to TEG is minimal compared to that from heat transfer^21,25^, highlighting that thermal effects dominate over viscous dissipation.

Figure 10d displays the variation of the Bejan number (Be) for the NFs at different Re. Higher Be values indicate that entropy generation is primarily due to heat transfer rather than fluid friction or internal irreversibility^15,21,23^. Finally, Table 4 provides the uncertainty analysis of the measurements conducted in this study, ensuring the reliability of the reported results.

**Table 4:** Uncertainty analysis of measurements.

Sl. no	Property	Maximum uncertainty (%)
1	Dynamic viscosity	2.4
2.	Thermal conductivity	1.8
3.	Reynolds number	3.2
4.	Heat flux	0.2
5.	Nusselt number	3
6.	friction factor	1.9

### Thermal performance factor (TPF)

**Fig. 11:**
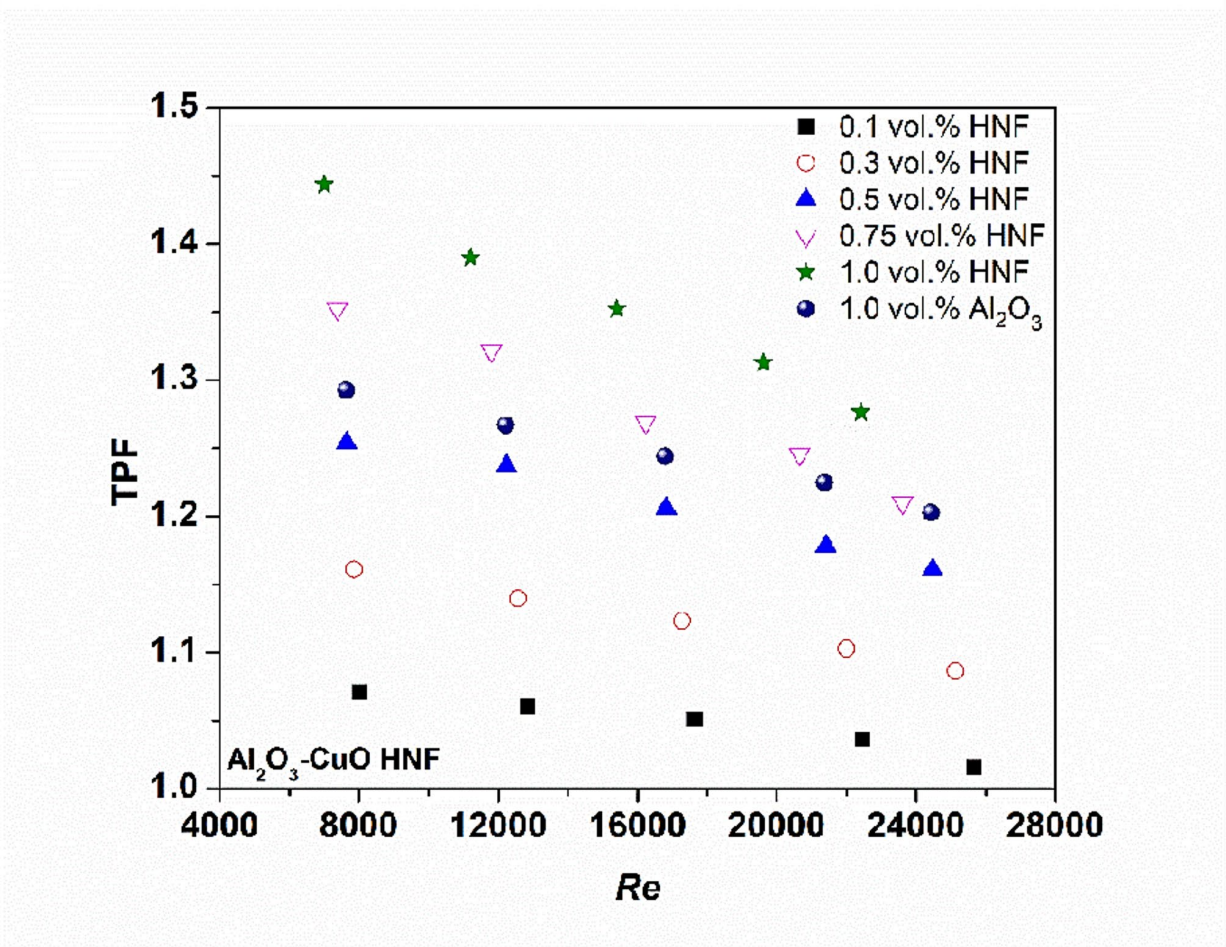
Thermal performance factor versus the Re.

The thermal performance factor (TPF) of NFs was calculated using Eq. (13)^26^, defined as the ratio of enhanced Nu to the increase in friction factor^25^. Figure 11 illustrates the TPF values for all tested NFs across the full Re range. A TPF value greater than 1 indicates that the fluid provides a net benefit in heat transfer performance. As shown, TPF exceeds 1 for all developed NFs, confirming their suitability as effective heat transfer media. Additionally, TPF increases with concentration, reflecting improved thermal performance. Notably, HNFs exhibit a higher TPF than Al₂O₃ NFs at 1.0 vol%, primarily due to the superior TC of the combined Al₂O₃–CuO system compared to the base fluid.

13 $$\:\text{T}\text{P}\text{F}=\:\left(\frac{{\text{N}\text{u}}_{\:\text{n}\text{f}}}{{\text{N}\text{u}}_{\text{b}\text{f}}}\right)/{\:\:\:\:\left(\frac{{\text{f}}_{\text{n}\text{f}}}{{\text{f}}_{\text{b}\text{f}}}\right)}^{1/3}$$

### Model-prediction with XGBoost

#### Development of prognostic models

**Fig. 12:**
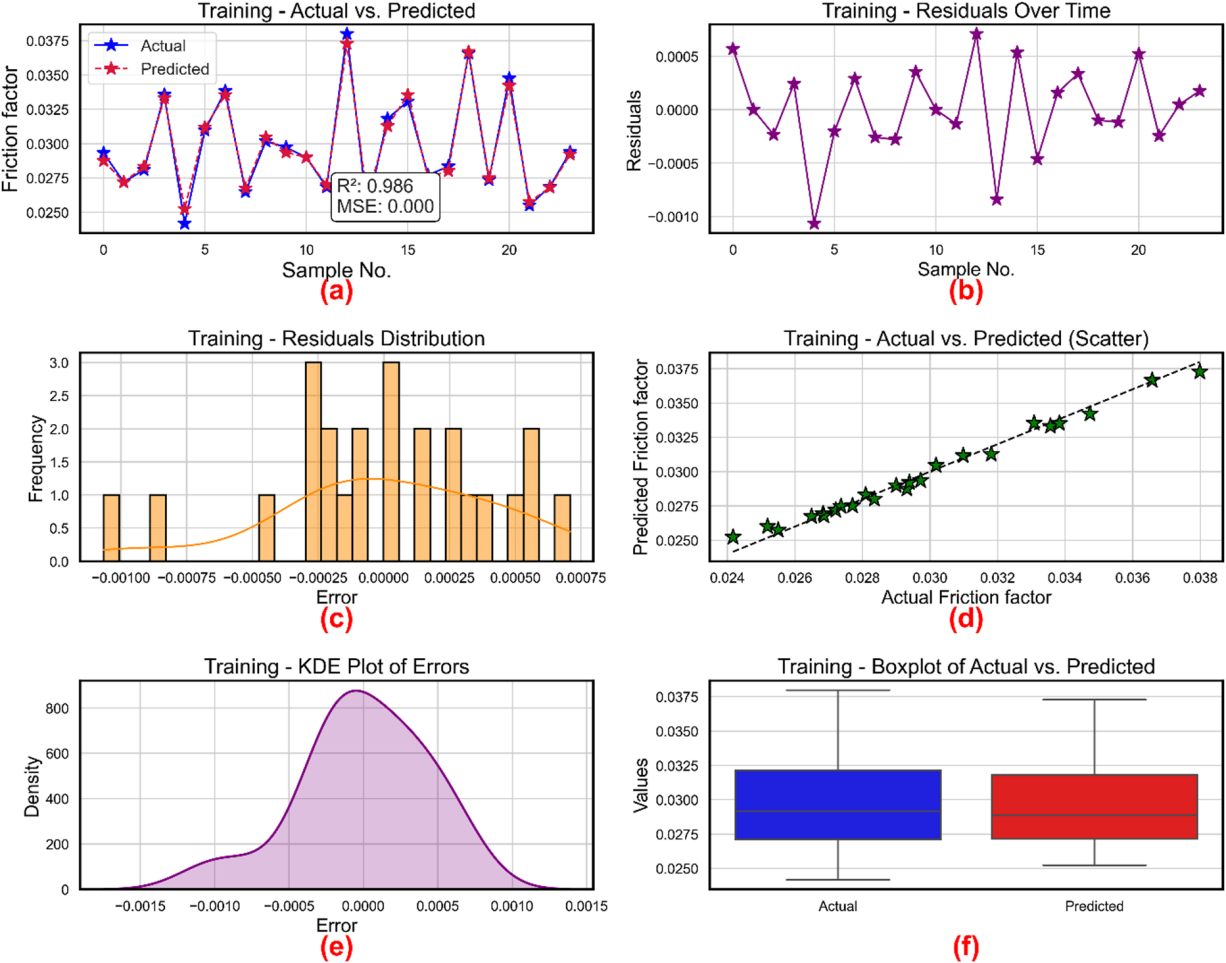
Friction factor model during training (**a**) actual vs. predicted values (**b**) residuals (**c**) distribution of residuals (**d**) scatter plot for actual vs. predicted values (**e**) kernel density estimation and (**f**) Boxplots.

XGBoost was employed for development of prognostic models for three critical parameters in NF behavior: Nu, friction factor, and TEG. Figure 12 shows the performance of the XGBoost based friction factor model during training phase. Table 5 lists the hyperparameters and their optimal values using a Bayesian method applied for hyperparameter optimization. Six diagnostic subplots (a–f) systematically show the performance assessment of the XGBoost based friction factor regression model, therefore exposing different statistical and visual insights on the accuracy and residual behavior of the model. Figure 12(a) shows for the training data across sample cases the actual and expected friction factor values. High model fidelity is shown by the predicted values (marked with red stars and dashed lines), which quite nearly track the actual values (blue markers with solid lines). With the coefficient of determination verifying that the model captures almost 98.6% of the variance in the friction factor, the provided statistical measures, R² = 0.686 and MSE = 0.000, represent almost flawless prediction accuracy. Figure 12b shows the residuals over sample counts, hence stressing the temporal pattern of prediction errors. The residual values show minimal bias and homoscedasticity by closely varying within the small range of around ± 0.001. The lack of a clear trend or structure across the samples implies that the model does not suffer from systematic mistakes, therefore confirming its dependability.

To examine their distribution, Fig. 12c shows the histogram of residuals. With most concentration around the center, the residuals are symmetrically scattered about zero. The almost normal distribution indicates that the XGBoost model did neither overfit nor underfit the training data, therefore supporting the presumption of error normality. Figure 12d shows projected against actual friction factor values a scatter plot. The neatly aligned data points along the diagonal (y = x), therefore supporting the robustness of the model even more. This alignment supports the R² value noted in sidebar Fig. 12a by showing the smallest variation between forecasts and actual data. The kernel density estimate (KDE) of prediction errors is shown in Fig. 12e, therefore approximating the residual distribution. The unimodal and centered at 0 KDE graphic supports the idea of minimal bias and verifies that the mistakes of the model are essentially random and symmetrically distributed. Figure 12f shows a boxplot comparison of actual and expected values. Both boxplots show identical medians and interquartile ranges; their whiskers span similar ranges, which suggests that the expected values well reflect the statistical characteristics of the real data. The symmetry and overlapping point to consistency in dispersion and central tendency. With well-behaved residuals and a great degree of statistical agreement between real and projected data, these subplots taken together show that the XGBoost model has attained accurate and dependable predictions of the friction factor.

**Fig. 13:**
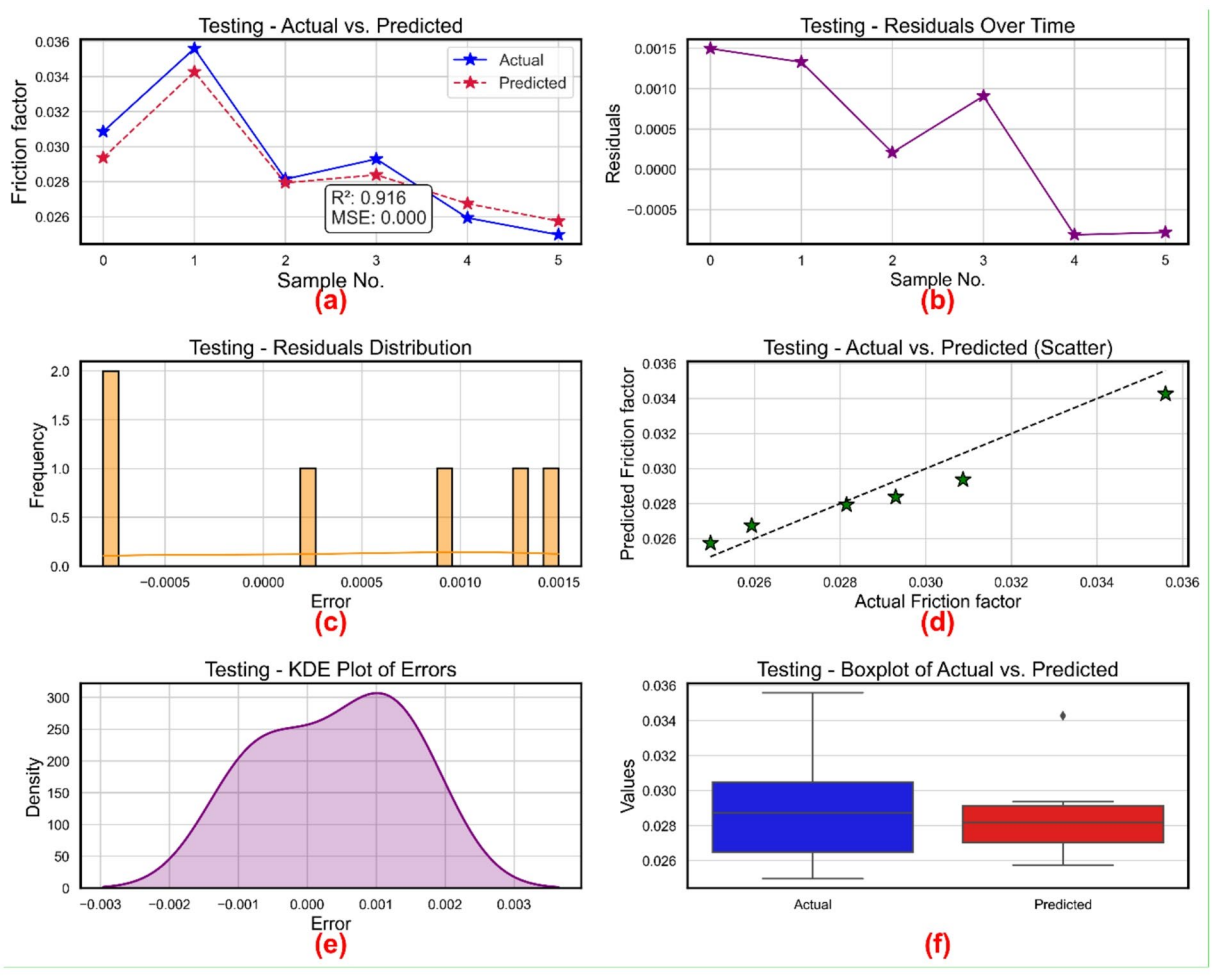
Friction factor model during testing (**a**) actual vs. predicted values (**b**) residuals (**c**) distribution of residuals (**d**) scatter plot for actual vs. predicted values (**e**) kernel density estimation and (**f**) Boxplots.

Figure 13 shows a comprehensive evaluation of the testing results for friction factor prediction applying several statistical and visual diagnostics. Figure 13a shows for six samples both the actual and expected friction factor values. With a high R² of 0.916 and a very low MSE of 0.000, the expected curve closely reflects the actual data, therefore indicating great prediction accuracy. Where values vary within the limited range of − 0.0006 to 0.0015, Fig. 13b displays the residuals—actual minus predicted—over the sample index. Random distribution of the residuals suggests the lack of systematic mistakes and supports model dependability.

Figure 13c shows residual histograms. Most residuals cluster close to zero, between − 0.0005 and 0.0015, hence verifying the tiny and symmetrically distributed prediction errors. Predicted against actual FF values, Fig. 13d displays the scatter plot. The data points closely to the 45° reference line, therefore supporting the little divergence between expected and actual values and a good connection. Figure 13e shows the KDE plot of residuals, therefore offering a smoothed picture of error density. The symmetric bell-shaped curve centered at zero and the peak around 0.001 emphasize how low mistakes are and how very regular their distribution is. Using boxplots, Fig. 13f also shows actual and expected friction factor values at last. Although expected values indicate somewhat smaller median and constricted range, both distributions have overlapping IQRs with one outlier in the actual collection.

All six subplots taken together support the model’s consistency in predicting friction factor during the testing phase, great accuracy, and resilience.

**Table 5:** Training hyperparameters range and optimized value.

Hyperparameter	Range	Friction factor	Nu	TEG
		Best value	Best value	Best value
n_estimators	50 to 500	248	456	106
max_depth	3 to 15	12	12	15
learning_rate	0.01 to 0.3	0.205055	0.296331	0.282944617
subsample	0.5 to 1.0	0.824576	0.666623	0.788317529
colsample_bytree	0.5 to 1.0	0.539745	0.933998	0.648353469
gamma	0.0 to 5.0	3.809672	2.874044	0.002257306
reg_alpha	0.0 to 5.0	0.011164	1.437054	0.080852028
reg_lambda	0.0 to 5.0	4.503554	3.396651	3.172687674

**Fig. 14:**
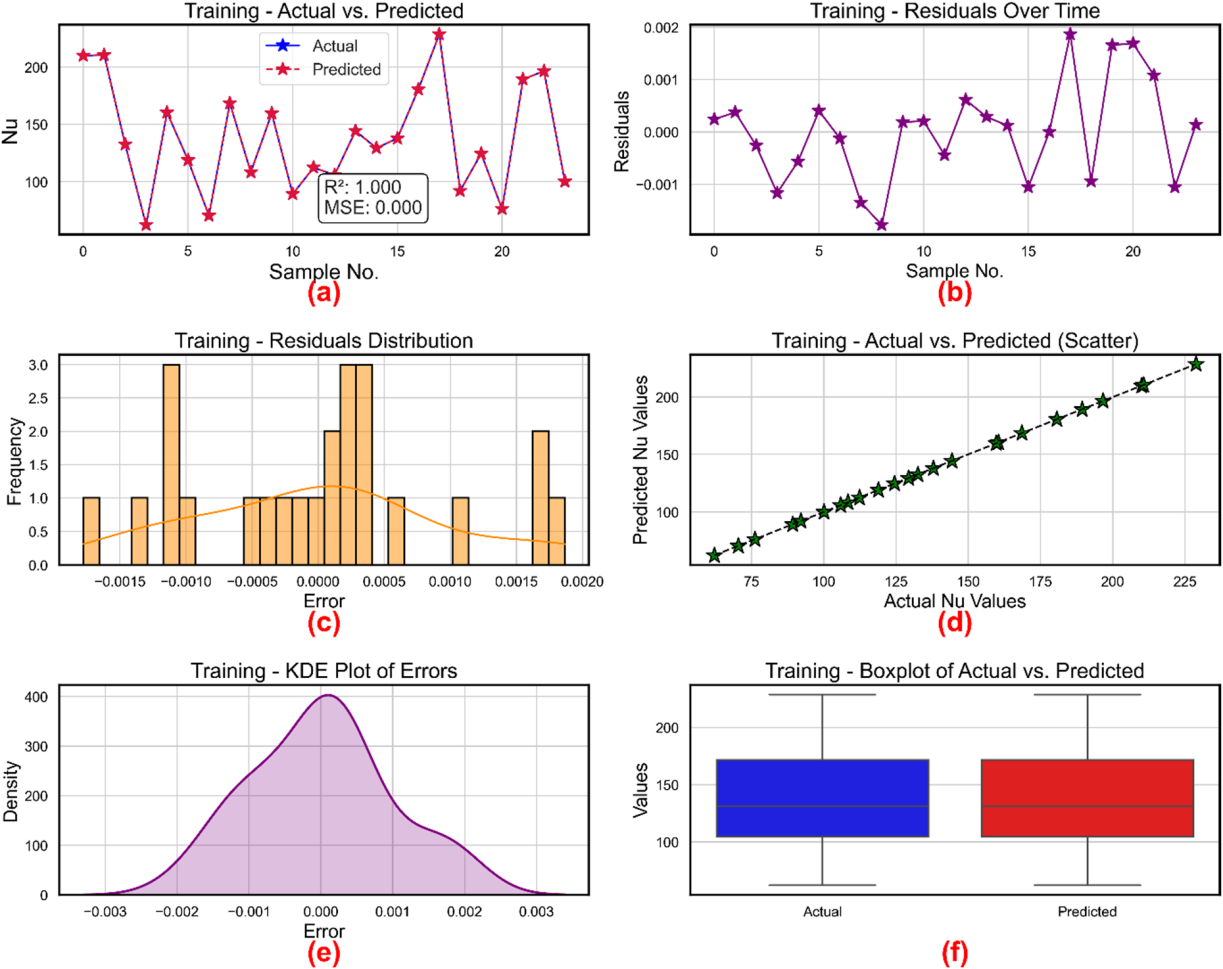
Nu prediction model during training (**a**) actual vs. predicted values (**b**) residuals (**c**) distribution of residuals (**d**) scatter plot for actual vs. predicted values (**e**) kernel density estimation and (**f**) Boxplots.

Figure 14 shows the XGBoost-based model’s training-phase assessment for Nu prediction. Bayesian approach for hyperparameters was used and the hyperparameters and their optimized values are listed in Table 4. Figure 14a shows, over six test samples, actual against expected Nu levels. Reflecting a good R² of 0.975 and a low MSE of 29.457, the predicted trajectory fits very well with the actual trend, therefore verifying the correctness of the model. Reiterating temporal stability in prediction accuracy, Fig. 14b shows the residuals ranging from around − 7.0 to + 7.5 across samples, thereby suggesting a tight error margin without clear drift. Figure 14c shows the residual histogram in which the mistakes are tightly spaced between − 6 and + 8. The figure shows the inclination of the model to generate symmetric low-magnitude errors devoid of dramatic deviations. Further confirming prediction authenticity and minimum bias, Fig. 14d displays a scatter plot of anticipated against real Nu values whereby all points rather nearly match the 45° line. A KDE graph of the prediction errors is given in Fig. 14e. Centered almost at zero, the error density shows a bell-shaped curve with most of the errors lying between − 15 and + 20. The symmetric and smooth curve implies that the model errors follow a quasi-normal distribution, therefore demonstrating a consistent and trustworthy learning pattern. Boxplots in Fig. 14f contrast the actual and anticipated Nu values. Their medians and IQRs are quite comparable. Though some minor outliers on both sides, the general agreement in spread guarantees stability in estimate. All the subplots in Fig. 14 together confirm the generalizing capacity, accuracy, and resilience of the XGBoost model for Nu prediction.

**Fig. 15:**
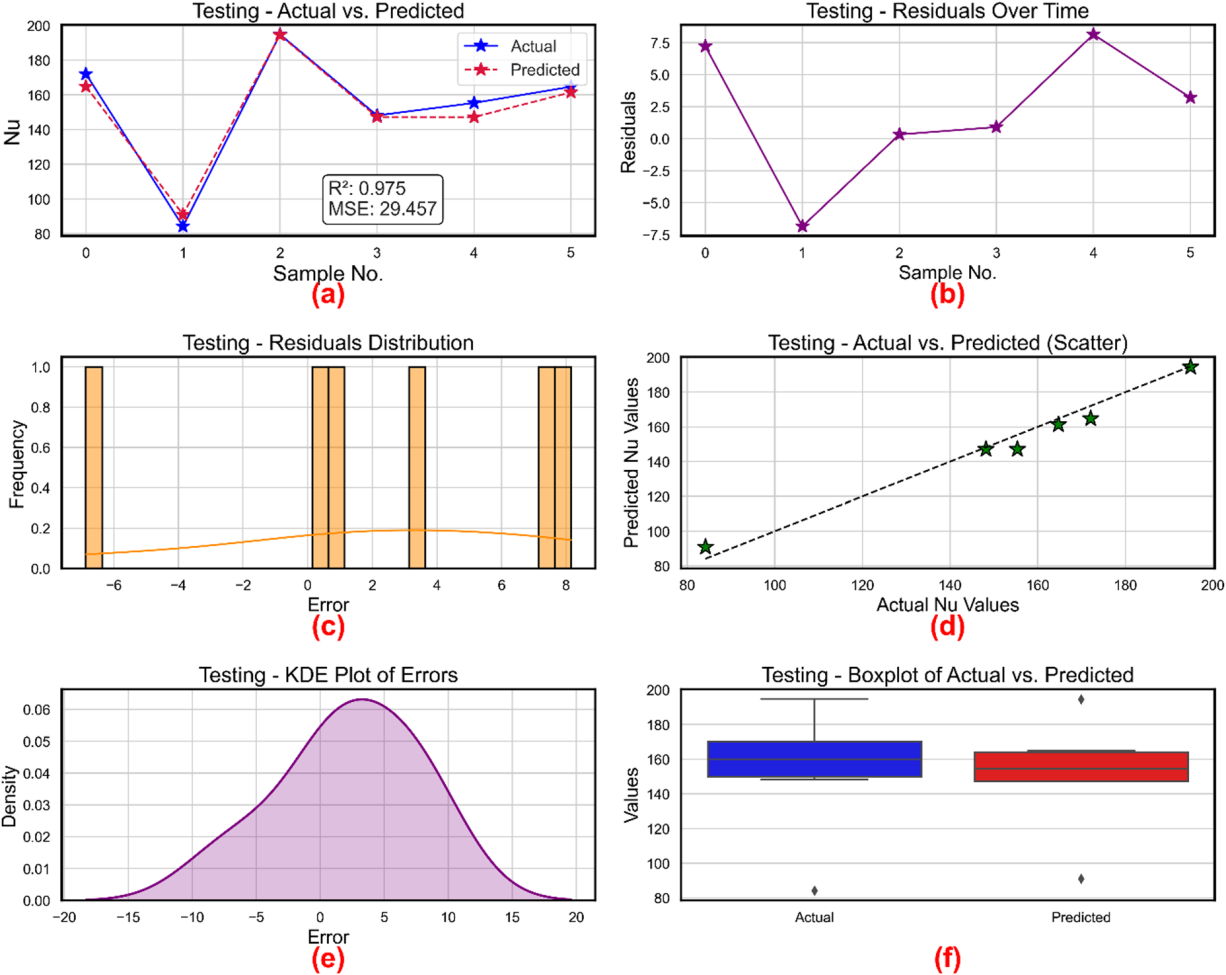
Nu prediction model during training (**a**) actual vs. predicted values, (**b**) residuals, (**c**) distribution of residuals, (**d**) scatter plot for actual vs. predicted values, (**e**) kernel density estimation and (**f**) Boxplots.

Figure 15 offers a picture of the testing performance of the XGBoost model for Nu estimation. With R² = 0.975 and MSE = 29.457, Fig. 15a shows robust alignment between actual and expected Nu values over six test samples. With great accuracy in model generalization, the expected trend essentially matches the actual curve. Plotting the residuals versus sample numbers, Fig. 15b oscillates between − 7 and + 7.5. Reflecting model neutrality and error stability, the dispersed and symmetric character of the residuals implies no obvious pattern or time-dependent bias. The residuals histogram in Fig. 15c fairly spans between roughly − 6 and + 8, therefore capturing the spectrum of prediction errors. The uniformly spaced frequencies confirm a very bell-shaped distribution with little skewness. Figure 15d shows a scatter plot contrasting expected from actual Nu values. Excellent predictive agreement shown by the alignment of data points along the reference line supports the ability of the model to approximate real values with minimum variance. Figure 15e shows a unimodal, symmetric profile centered on zero by including the KDE plot for residuals. Most mistakes fall under the ± 20 range, implying that the prediction errors of the model are primarily friction factor. These visual diagnostics confirm the test prediction dependability and resilience of the XGBoost model.

**Fig. 16:**
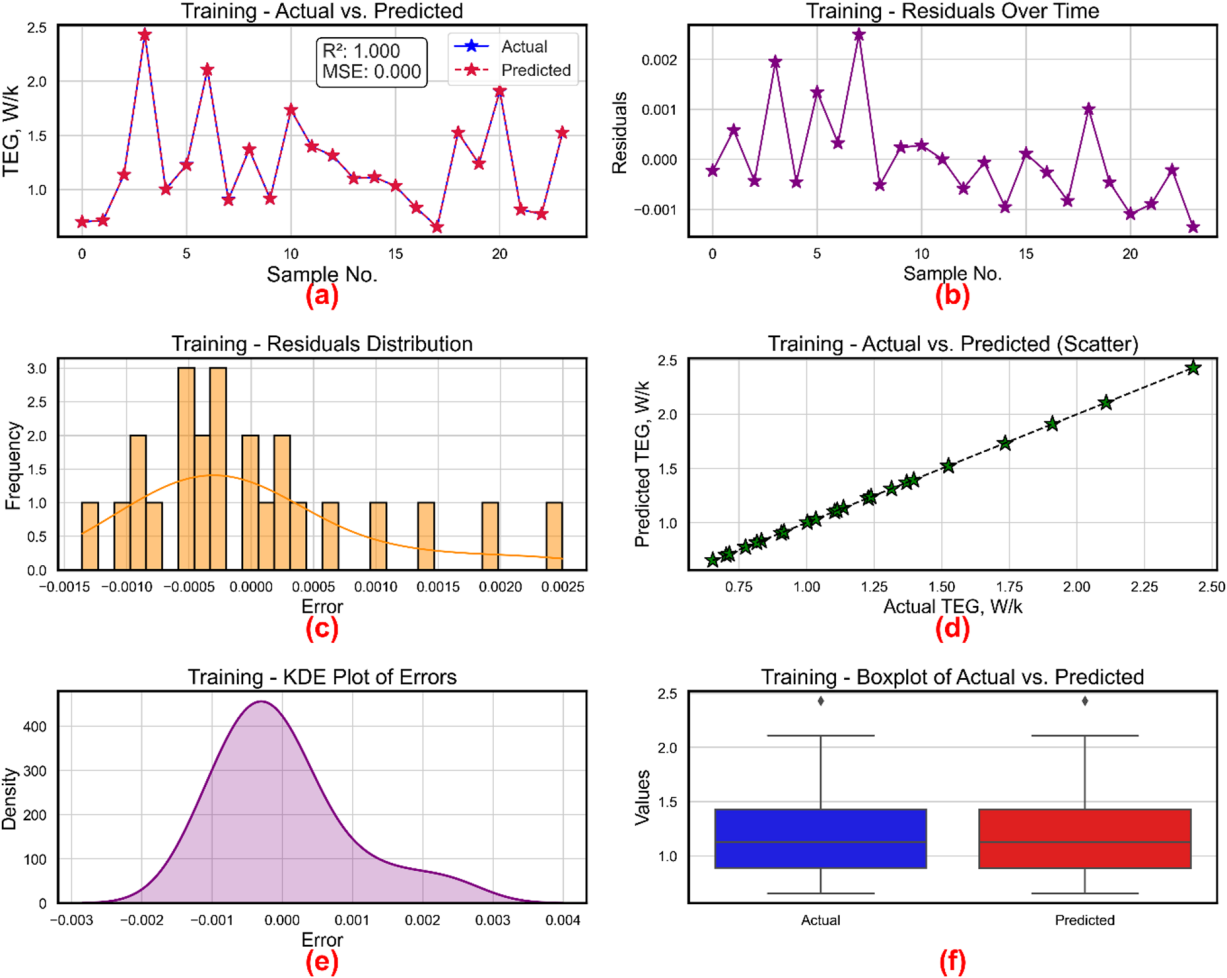
TEG prediction model during training (**a**) actual vs. predicted values, (**b**) residuals, (**c**) distribution of residuals, (**d**) scatter plot for actual vs. predicted values, (**e**) kernel density estimation and (**f**) Boxplots.

Figure 16 shows methodically how the XGBoost model performs during the training phase to forecast TEG. With R² = 1 and almost nil MSE, the actual and predicted TEG values are practically exactly superposed as shown in Fig. 16a, hence proving an excellent match and faultless learning during training. Residuals are shown in Fig. 16b versus the sample index. These tiny values, which vary symmetrically around zero, point to a lack of systematic error and validate the model’s accuracy in faithfully copying training data free from drift or overfit. Figure 16c shows residuals’ histogram. Most mistakes cluster close to zero with some right skewness. The limited distribution suggests that deviations from the real values are rather modest, therefore validating the great learning fidelity of the model. Figure 16d a scatter plot comparing expected and actual TEG values. Tightly aligned along the reference diagonal line, the data points show almost one-to-one connection between the expected outputs and the real values. Figure 16e display error kernel density estimate. About − 0.0008, a thick peak shows up with a smooth tapering on both sides. The symmetrical and compact form suggests that extremely low-magnitude errors prevail, and normal distribution of the residuals is indicated. Finally, Fig. 16f shows the actual TEG value boxplots against expected ones. Both distributions show same medians, interquartile ranges, and outliers. This supports the preceding results showing statistically compatible forecasts of the model with the training data.

**Fig. 17:**
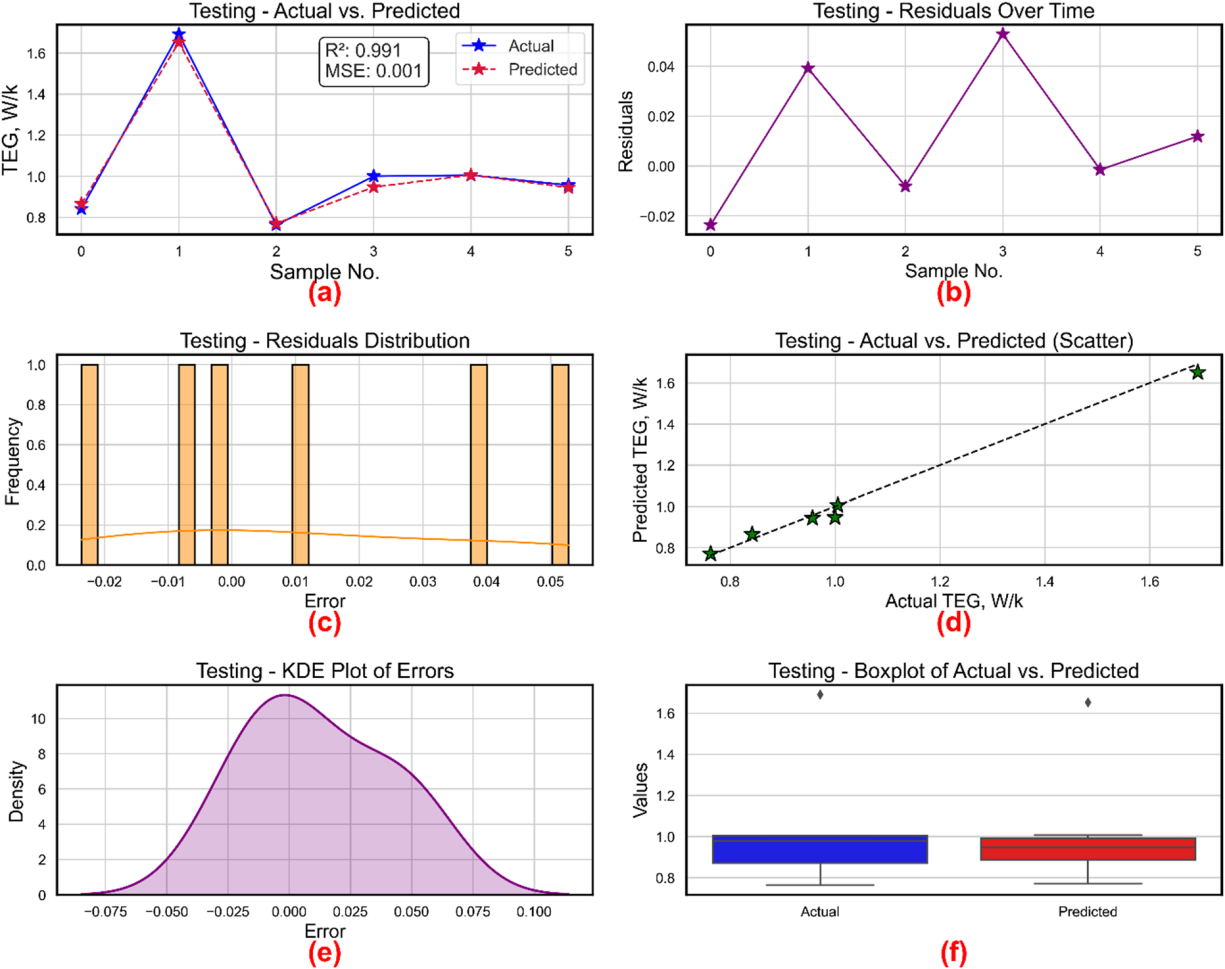
TEG prediction model during training (**a**) actual vs. predicted values, (**b**) residuals, (**c**) distribution of residuals, (**d**) scatter plot for actual vs. predicted values, (**e**) kernel density estimation and (**f**) Boxplots.

Six subplots in Fig. 17, each offering unique perspectives on the accuracy and error behavior of the XGBoost-based model for estimating TEG, show its testing performance. Further substantiated by a R² of 0.991 and a very low MSE of 0.001, Fig. 17a displays the line plot of real vs. projected TEG values across six test samples, where the two curves nearly overlap demonstrating great agreement. Plotting the residuals over time in Fig. 17b, one can see that mistakes remain minor and vary just little about zero; the greatest residual is somewhat over 0.04. The histogram of residuals shown in Fig. 17c shows a compact, mainly symmetric distribution centered around zero, hence suggesting little bias and well-distributed errors. Further verifying the great predictive accuracy of the model, Fig. 17d the scatter plot of predicted against real TEG values reveals points firmly aligned along the 45-degree reference line. With most of the mistakes falling between − 0.075 and 0.1, Fig. 17e shows the KDE plot of errors using a smooth, bell-shaped curve centered at zero, hence verifying the assumption of normality in residual distribution. Finally, Fig. 17f is a boxplot contrasting actual from projected values. Though a few outliers on the top end, the general distribution and spread of actual and projected values are very matched in both boxes in range and median. All six subplots taken together show that the XGBoost model predicts TEG values with great accuracy, low error, and good generalizing capability.

### Correlations

An empirical correlation for the Nu and friction factor was developed by analyzing the influence of Re, Prandtl number, temperature, and concentration in dimensionless form. Temperature was normalized to a reference of 60 °C, and volume concentration was scaled as a percentage. A logarithmic transformation was applied to linearize the relationship, and multiple linear regression was performed using Microsoft Excel to determine the optimal constants and exponents. The resulting correlation reliably predicts convective heat transfer performance in HNFs across a range of flow and thermal conditions.

The correlations are developed to determine the Nu and friction factor of the Al_2_O_3_-CuO (50:50) HNF based on the data acquired through testing presented in Eqs. (14) and (15).

For Nu,

14 $$\:\text{N}\text{u}\hspace{0.17em}=\hspace{0.17em}0.11\times\:{\text{R}\text{e}}^{0.741}\times\:{\:\text{P}\text{r}}^{-0.413}{{\:\times\:\left(\frac{\text{T}}{60}\right)}^{-0.56}\times\:\left(1.0+\frac{{\upphi\:}}{100}\right)}^{51.8}$$

Average and standard deviations are 3.06 and 4.01%, respectively; R^2^ = 0.97.

For friction factor,

15 $$\:\text{f}\hspace{0.17em}=\hspace{0.17em}0.21\times\:{\text{R}\text{e}}^{-0.217}\:\times\:{\text{P}\text{r}}^{0.102}{{\:\left(\frac{\text{T}}{60}\right)}^{0.13}\times\:\left(1.0+\frac{{\upphi\:}}{100}\right)}^{13.8}$$

Average and standard deviations are 2.4 and 3.0%, respectively, R^2^ = 0.984.

Valid for, 7000 ≤ Re ≤ 26,444, 5.33 ≤ Pr ≤ 5.7, and 0.1 ≤ vol% ≤ 1.0.

## Conclusions

Numerical and experimental studies were conducted to investigate the hydrodynamic performance and entropy generation of Al₂O₃–CuO (50:50) HNFs under constant heat flux conditions across various Reynolds numbers. The experimentally obtained data were further used to develop predictive models using the XGBoost algorithm. The key conclusions drawn from this study are:

1. HNF exhibited a maximum enhancement in HTC of 23.6% over Al₂O₃ NFs at 1 vol%.
2. The maximum increase in Nusselt number was 51% for HNF and 32.1% for Al₂O₃ nanofluids compared to water, at 1 vol%.
3. The highest-pressure drop of 24.3% for HNF and 14.2% for Al₂O₃ NFs relative to water, at 1 vol%.
4. The friction factor of HNF was 7.7% higher than that of Al₂O₃ nanofluids at 1 vol%.
5. TPF reached 1.44 for HNF and 1.29 for Al₂O₃ nanofluids at 1 vol%, indicating favorable thermal-hydraulic performance.
6. The XGBoost model demonstrated excellent accuracy in predicting total entropy generation with a test R² of 0.991 and a minimal MSE of 0.001.
7. Friction factor predictions were reliable, with training and test R² values of 0.686 and 0.916, respectively, and low associated MSE.
8. While Nusselt number predictions showed some variation, the model still achieved good test accuracy (R² = 0.975, MSE = 29.457), confirming the applicability of XGBoost in modeling nonlinear thermal systems for energy optimization.

Overall, the study provides new insights into the thermohydraulic performance of NFs and demonstrates the potential of machine learning-based prognostic models. Future research can explore the use of these NPs in high-temperature fluids like thermal oils for advanced energy systems, temperature-dependent thermophysical properties will be considered for simulation to better reflect real operating conditions. Additionally, long-term applications demand further exploration of cost, stability, and optimal nanoparticle concentration.

## Data Availability

The datasets during and/or analyzed during the current study are available from the corresponding author upon reasonable request.

## Abbreviations

BO: Bayesian optimization
CFD: Computational fluid dynamics
GPR: Gaussian process regression
HNF: Hybrid nanofluid
HTC: Heat transfer coefficient, wm^−1^ k^−1^
HTF: Heat transfer fluid
KDE: kernel density estimate
MAE: Mean absolute error
mL: Milli liter
MSE: Mean squared error
mV: Milli volts
NF: Nanofluid
NP: Nanoparticle
Nu: Nusselt number
RBF: Radial basis function
Re: Reynolds number
RMSE: Root mean squared error
SS: Stainless steel
SDBS: Sodium dodecylbenzene sulfonate
TC: Thermal conductivity, Wm^−1^ K^−1^
TEG: Total entropy generation, W K^−1^
TPF: Thermal performance factor
VST: Viscosity, mPa.s

## List of symbols

A_s_: Test section surface area, m^2^
Be: Bejan number
c_p_: Specific heat, J kg^−1^. K^−1^
D: Tube diameter, m
f: Friction factor
h: Heat transfer coefficient, W m^−1^ K^−2^
I: Current, amperes
*I*: Turbulent intensity
k: Thermal conductivity, Wm^−1^ K^−1^
L: Tube length, m
m: Mass flow rate, kg s^−1^
Pr: Prandtl number, $\:\text{P}\text{r}=\frac{\mu\:\:{c}_{p\:}}{{k}_{\:}}\:\:$
$\:\varDelta\:p$: Pressure drop, Pa
Q: V$$\:\times\:$$I, Watt
R^2^: Coefficient of determination
Re: Reynolds number, $\:\text{R}\text{e}=\frac{\rho\:vD}{\mu\:}\:\:$
T_b_: Fluid bulk temperature, K
T_o_: Fluid outlet temperature, K
T_i_: Fluid inlet temperature, K
T_s_: Surface temperature, K
V: Voltage, volts
*V*: Fluid velocity, ms^−1^

## Greek symbols

*µ*: Dynamic viscosity, mPa.s
*ρ*: Density, kg m^−3^
*φ*: Volume concentration, %

## Subscripts

Bf: Base fluid
Nf: Nanofluid
Np: Nanoparticle
